# First-in-human study of ^99m^Tc-labeled fucoidan, a SPECT tracer targeting P-selectin

**DOI:** 10.1186/s13550-024-01173-8

**Published:** 2024-11-19

**Authors:** Reindert F. Oostveen, Kang H. Zheng, Yannick Kaiser, Nick S. Nurmohamed, Jeffrey Kroon, Tim C. de Wit, Edwin Poel, Joel Aerts, Francois Rouzet, Erik S. G. Stroes, Didier Letourneur, Hein J. Verberne, Cédric Chauvierre, Mia R. Ståhle

**Affiliations:** 1 Department of Vascular Medicine, Amsterdam Cardiovascular Sciences, Amsterdam UMC, University of Amsterdam, Amsterdam, The Netherlands; 2 Department of Cardiology, Amsterdam UMC, University of Amsterdam, Amsterdam, The Netherlands; 3 Department of Experimental Vascular Medicine, Amsterdam UMC, University of Amsterdam, Amsterdam, The Netherlands; 4 Amsterdam Cardiovascular Sciences, Atherosclerosis & Ischemic Syndromes, Amsterdam, The Netherlands; 5 Laboratory of Angiogenesis and Vascular Metabolism, VIB-KU Leuven Center for Cancer Biology, VIB, 3000 Louvain, Belgium; 6 Laboratory of Angiogenesis and Vascular Metabolism, Department of Oncology, KU Leuven and Leuven Cancer Institute (LKI), 3000 Louvain, Belgium; 7 Department of Radiology and Nuclear Medicine, Amsterdam UMC, University of Amsterdam, Amsterdam, The Netherlands; 8 Nuclear Medicine Department, Bichat Hospital, APHP, Paris, France; 9 UMR-S U1148 INSERM, Laboratory for Vascular Translational Science (LVTS), Université Paris Cité, Université Sorbonne Paris Nord, 75018 Paris, France; 10 Fédération de Recherche en Imagerie Multi-Modalité (FRIM), Université Paris Cité, UMS 34, 75018 Paris, France

**Keywords:** Dosimetry, SPECT, Thrombus, P-selectin, Fucoidan

## Abstract

**Background:**

Activation of endothelial cells and platelets in atherothrombosis is characterized by upregulation of P-selectin. As a consequence, P-selectin represents a potential target for molecular imaging to identify thrombosis at an early stage. Fucoidan is a polysaccharide ligand extracted from brown algae with nanomolar affinity for P-selectin. This first-in-human study evaluated in healthy volunteers the safety, whole-body biodistribution, and dosimetry of ^99m^Tc-fucoidan (Good Manufacturing Practices grade). We also investigated whether we could observe binding of ^99m^Tc-fucoidan to human thrombi ex vivo and in vivo. In ten healthy volunteers, conjugate whole-body scans were performed up to 24 h following intravenous injection of ^99m^Tc-fucoidan (370 MBq). Moreover, ^99m^Tc-fucoidan uptake in ex vivo human thrombi (n = 11) was measured by gamma counting. Additionally, three patients with a newly diagnosed deep vein thrombosis (DVT) were subjected to ^99m^Tc-fucoidan SPECT/CT imaging.

**Results:**

^99m^Tc-fucoidan was well tolerated in all participants without any drug-related adverse events. The total-body absorbed dose in males was comparable to females (0.012 ± 0.004 vs. 0.011 ± 0.005 mSv/MBq; *p* = 0.97). Gamma counting experiments demonstrated binding of tracer to ex vivo human thrombi that was 16% lower after blocking with a natural P-selectin ligand, Sialyl Lewis X. ^99m^Tc-fucoidan demonstrated specific uptake at the thrombus site in one out of three scanned patients with DVT.

**Conclusions:**

^99m^Tc-Fucoidan has a favorable biodistribution and safety profile. ^99m^Tc-fucoidan exhibited specific binding to human thrombi in both in vivo and ex vivo settings. Nonetheless, the in vivo results do not support further clinical investigation of ^99m^Tc-fucoidan as an imaging modality for DVT. Other clinical implementations of a technetium− 99m-labeled P-selectin tracer should be considered.

*Trial registration*: Clinicaltrials,NCT03422055.Registered 01/15/2018. URL: https://clinicaltrials.gov/study/NCT03422055Landelijk Trial Register, NL7739. Registered 4/2/2019 . https://onderzoekmetmensen.nl/en/trial/26785

**Supplementary Information:**

The online version contains supplementary material available at 10.1186/s13550-024-01173-8.

## Introduction

Cardiovascular disease is hallmarked by endothelial dysfunction contributing to a pro-inflammatory state within the arterial wall [1]. As inflammation is a key process throughout all stages of atherogenesis, there is a strong interest to develop non-invasive imaging modalities to assess arterial wall inflammation, in particular for the evaluation of novel anti-inflammatory agents [2]. The inflammatory pathway in atherothrombosis is characterized by a complex of interactions between endothelial cells, leukocytes, and platelets. P-selectin is an adhesion molecule expressed at the surface of activated platelets and endothelial cells [3]. Platelet P-selectin interacts with leukocytes to form stable platelet-leukocyte aggregates during the early phase of thrombogenesis, while endothelial P-selectin mediates leukocyte adhesion, rolling, and extravasation of mononuclear inflammatory cells into the subendothelial space. Thus, P-selectin represents a potential molecular imaging target for early detection of atherothrombosis.

Molecular imaging of P-selectin by means of Positron Emission Tomography (PET), Single-Photon Emission Computed Tomography (SPECT), ultrasound, and Magnetic Resonance Imaging (MRI) have been investigated in several animal models of cardiovascular disease, including atherosclerosis [4], ischemic stroke [5], venous thromboembolism [6], cardiac thrombi [7], and abdominal aortic thrombosis/aneurysms [8–11]. In these studies, imaging agents formulated with antibodies against P-selectin or synthetic mimics of Sialyl Lewis X (SLe^x^), a natural ligand of P-selectin, detected upregulation of P-selectin in sites of acute atherothrombotic events. However, human data is lacking to date. Previously, we identified fucoidan as a polysaccharide ligand with nanomolar affinity for P-selectin [12]. Fucoidan is a sulfated polyfucose extracted from brown algae and is a natural mimic of SLe^x^. Technetium− 99m (^99m^Tc)-labeled fucoidan selectively binds to P-selectin expressed by activated human platelets in vitro, and it enables SPECT/CT to visualize platelet-rich thrombi in animal models of abdominal aortic aneurysm and endocarditis as well as endothelial activation in murine ischemia–reperfusion injury [13].

Recently, we developed ^99m^Tc-fucoidan as a Good Manufacturing Practice (GMP)-grade clinical imaging agent [14]. In the present study, we evaluated the safety profile, whole-body biodistribution, and estimated absorbed radiation doses of this new radiopharmaceutical in humans. Moreover, we provide proof-of-principle by performing experiments on human thrombi with ^99m^Tc-fucoidan ex vivo*.* Three patients with a newly diagnosed deep vein thrombosis (DVT) underwent ^99m^Tc-fucoidan SPECT/CT.

## Methods

### Study design for phase I

This study had an open-label design to assess the safety, whole-body biodistribution kinetics, and dosimetry of ^99m^Tc-fucoidan in 10 healthy volunteers consisting of 5 male and 5 female subjects. ^99m^Tc-fucoidan was synthesized according to GMP guidelines as described previously [14] and produced by Eurofins Amatsi Group (Fontenilles, France). The molar activity of ^99m^Tc-fucoidan was 2994 ± 872 MBq/nmol at the end of synthesis, and the radiochemical purity exceeded 95% in every batch. Healthy subjects were intravenously injected with 356 ± 14 MBq of ^99m^Tc-fucoidan, after undergoing baseline examinations. Whole body planar scintigraphy was performed at multiple time points after radiotracer administration (t = 30 min, 1.5 h, 3 h, 6 h and 24 h) and SPECT imaging at t = 2 h, based on previously reported blood clearance in a murine study [13]. Blood and urine analyses were performed before and after imaging.

The study was conducted in accordance with the Declaration of Helsinki and in compliance with current Good Clinical Practice guidelines. The protocols were approved by the local institutional review board, and all participants provided written informed consent. The study was registered as a clinical trial (NCT03422055, NL6703).

### Whole-body scintigraphy

Planar whole-body scans were acquired with an energy peak at 140 keV, 15% energy window, table speed 8 cm/min, without automatic body contour and a matrix size of 256 × 1024 using low energy high resolution collimators. Dual-head SPECT was performed with the same energy peak, energy window and collimators as used for the whole-body scans with a matrix size of 128 × 128, 32 projections per detector; 40 s per angle, step mode and a noncircular rotation of the chest and the abdomen. All scans were performed on a Siemens Symbia-S.

### Distribution kinetics and radiation dose estimates

All planar images were analyzed by drawing regions of interest for target organs using a dedicated software package (Olinda/EXM version 2.0, Hermes Medical Solutions, Stockholm, Sweden) [15, 16]. Whole-body scan derived values were used to determine biodistribution, residence times and dosimetry of ^99m^Tc-fucoidan. Estimated organ doses were calculated from the geometric mean images, using the ICRP 89 Adult Male and Female phantom models in OLINDA/EXM. All biodistribution results are expressed as a percentage of the injected dose. A calibration source was used to convert the measured counts into the percentage injected dose activity. The same dose calibrator was used for the creation of the calibration source and for assay of injected subject doses. SPECT data were not used for biodistribution and dosimetry analysis. No attenuation correction was applied for these analyses.

The well counter used has a yearly calibration and validation of the most used radiotracers. In addition, we added for each subject an appropriate standard ^99m^Tc activity to the measurements of the collected blood and urine samples obtained at different time intervals.

Regions of interest (ROIs) were drawn around the organs of interest to quantify tracer uptake [17]. The strategies we adopted to limit overlapping organs from distorting tracer uptake measurements can be found in the Supplemental Information. Whole-body region and bladder ROI were used to determine urinary excretion.

### Safety assessment

Clinical assessment including vital signs and electrocardiography was performed before each imaging acquisition. Healthy volunteers were continuously monitored for adverse events. Blood samples were collected and analyzed. Analyses included hematology, hemostasis, and chemistry panels.

### Blood and urine measurements

Urinary and venous blood samples were collected before and after ^99m^Tc-fucoidan injection at set intervals. Urine samples were collected for 24 h, in the following intervals: 0–1, 1–3, 3–6 and 6–24 h after injection of ^99m^Tc-fucoidan. Venous blood samples were collected in the following intervals: 0.5, 1.5, 3, 6, and 24 h after injection of ^99m^Tc-fucoidan.

The radioactivity of whole blood was measured with a gamma counter (Wizard 2480, Perkin Elmer). Plasma was separated by centrifugation (3,000 g for 15 min at 4 °C), radioactivity was measured and decay-corrected for the time of injection. The total urine volume was measured, and a 1 mL sample was measured with a gamma counter. Radioactivity measurements were corrected for total volume of urine and duration of time intervals. The gamma counting measurements were done in three duplicate 1 mL samples of the collected blood and urine at different time intervals combined with an appropriate standard ^99m^Tc activity to convert from counts to MBq.

### Ex vivo binding study

To study ^99m^Tc-fucoidan uptake in a disease-like setting, we incubated ex vivo human thrombus specimens with the tracer in the presence or absence of an excess amount of competitive binder. Venous blood samples were withdrawn from 11 healthy volunteers (6 males and 5 females, mean age 28.0 ± 1.8 years) into clot activator tubes (BD Vacutainer) and centrifuged for 15 min (3,810 g at 20 °C) to induce thrombus formation. Thereafter, serum was discarded and remaining thrombus was cut into cylinders of approximately 3 mm in thickness. Two cylinders per individual were used. As a positive control, the thrombus was incubated with 0.1 MBq of ^99m^Tc-fucoidan (1.5 mL, tracer solution 0.07 MBq/mL) for 60 min at room temperature (RT). To assess the specificity of tracer binding, 1 µg (100 µL) of 3’-Sialyl-Lewis-X-tetrasaccharide (SLe^x^, S1782, Sigma-Aldrich) was added two minutes before ^99m^Tc-fucoidan in a parallel thrombus. The dose of SLe^x^ was calculated to be a 150-fold amount compared with the ^99m^Tc-fucoidan. After incubation, the tracer solutions were discarded, and the thrombi were washed twice with 1.5 mL of phosphate-buffered saline (Fresenius Kabi, Netherlands). The radioactivity was measured using a gamma counter (2480 Wizard, Perkin Elmer) and radioactivity values were normalized for decay and weight of the thrombus. Results are depicted as counts per second/gram (CPS/g).

### Deep vein thrombosis assessment

We studied whether ^99m^Tc-fucoidan also binds to thrombi in vivo. This preliminary Phase IIa study was approved by the local institutional review board, and all participants provided written informed consent. The study was registered as NL7739. Thus, 3 patients with deep vein thrombosis (DVT), as diagnosed by routine diagnostic compression ultrasound sonography, were subjected to ^99m^Tc-fucoidan SPECT imaging. All scans were performed within 48 h after treatment initiation. The patient cohort comprised individuals aged 50, 55, and 60 years, two of them being female.

### Statistical analysis

Data are represented as means with standard deviations. To assess the difference in uptake for males and females an unpaired Student’s t-test was performed. For repeated measures data, we performed one-way ANOVA or mixed-model analysis. Statistical analyses were performed using the SPSS statistics software (Version 26, IBM). A *p* value of < 0.05 was considered statistically significant.

## Results

### Baseline characteristics

Baseline characteristics of 10 healthy volunteers are presented in Table 1. At screening, the mean age was 64 ± 7 years, the average blood pressure was 131 ± 9/80 ± 8 mmHg, and the mean BMI was 26.5 ± 4.8 kg/m^2^. Four subjects were ex-smokers and one was actively smoking. All subjects had normal liver and renal function tests. They did not use any concomitant medication.

**Table 1:** Baseline characteristics

	Study participants (n = 10)
Patient characteristics	
Age (years)	63.5 ± 7.2
Sex (male)	5 (50)
Smoker (yes)	1 (10)
Body mass index (kg/m^2^)	24.5 [22.7, 29.4]
Systolic blood pressure (mmHg)	132 [126, 140]
Diastolic blood pressure (mmHg)	76 [73, 82]
Laboratory parameters	
C-reactive protein (mg/L)	0.8 [0.5, 1.1]
White blood cell count (× 10^9/L)	5.4 [4.1, 5.6]
Thrombocytes (× 10^9/L)	201 [176, 249]
Creatinine (μmol/L)	82 [77, 85]
Estimated glomerular filtration rate (mL/min/1.73m^2^)*	75 [71, 81]
Alanine aminotransferase (U/L)	18 [16, 23]
Aspartate transaminase (U/L)	22 [19, 23]

Values are presented as mean ± standard deviation, median [interquartile range] or number (percentage). *Calculated using the Chronic Kidney Disease Epidemiology Collaboration (CKD-EPI) equation

### Safety and tolerability

^99m^Tc-fucoidan was well tolerated by all participants without any drug-related adverse event. During the study, there were no clinically significant changes in blood pressure or heart rate, on electrocardiography. Clinical chemistry before tracer administration is shown in Table 1.

### Whole-body biodistribution and radiation dosimetry

**Fig. 1:**
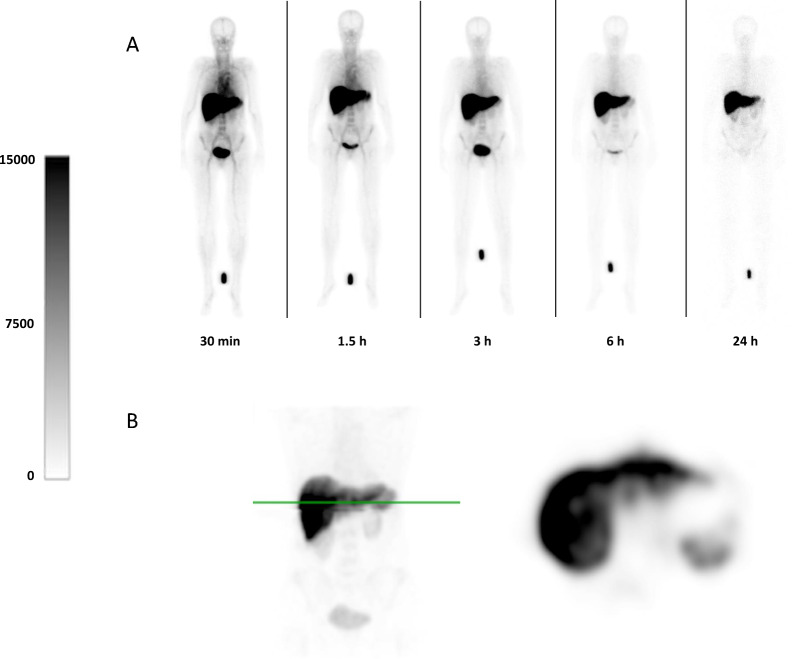
Whole body scintigraphy and SPECT (**A**) Representative whole body anterior planar scintigraphy of ^99m^Tc-fucoidan administered to a healthy volunteer. A reference standard was placed between the legs. Planar images are through 24 h. The scale bar is measured in absolute counts. **(B)** SPECT images of the thorax and abdomen at 2 h after injection. Liver, spleen and kidneys are visible on a maximum intensity projection of frontal plane view (left panel), with the green line indicating the position of the corresponding axial slice (right panel)

Typical examples of anterior images are depicted in Fig. 1A. Physiological uptake was visualized in the liver, kidney, spleen, and bladder. These observations were confirmed by the thoracic and abdominal SPECT (Fig. 1B).

**Fig. 2:**
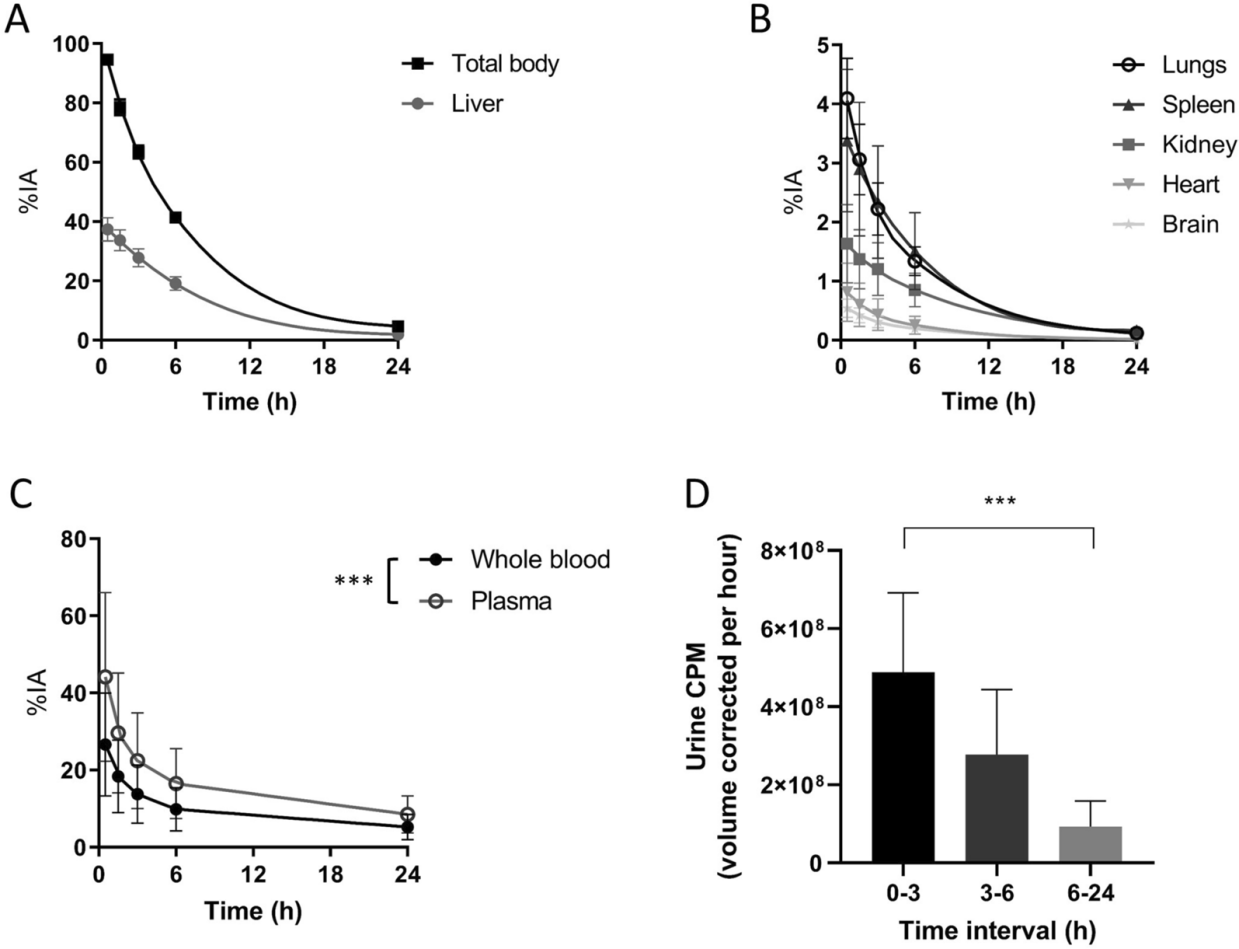
Biodistribution of ^99m^Tc-fucoidan Time activity curves of **(A)** total body, liver and **(B)** other organs as determined using planar scintigraphy. **(C)** Radioactivity in whole blood and plasma samples were measured using a well counter and decay-corrected for the time of injection. **(D)** Urine samples were measured and corrected for total volume of urine collected and duration of time intervals

The injected radioactivity was cleared rapidly with a total body activity of 4.7 ± 0.7% remaining after 24 h (Fig. 2A). Substantial activity was observed in the liver, which decreased from 37.3 ± 5.1% at 30 min after injection to 19.1 ± 3.0% after 6 h. Low activity (< 5%) was observed in other organs (Fig. 2B). In line, blood pool and plasma activity diminished quickly after the first 6 h (Fig. 2C). Plasma activity levels were significantly higher than whole blood levels in all time points (the plasma-to-blood ratio at 0.5, 1.5, 3, 6, and 24 h was 1.67, 1.64, 1.70, and 1.69 respectively, *p* for trend < 0.001). Radioactivity in urine was the highest in the first time interval and decreased significantly over the subsequent intervals (t = 0–3: 4.11 × 10^8^ ± 2.41 × 10^8^ CPM/hour; t = 3–6: 2.67 × 10^8^ ± 1.60 × 10^8^ CPM/hour; t = 6–24: 4.35 × 10^7^ ± 3.23 × 10^7^ CPM/hour; *p* for trend < 0.001). Despite explicit instructions, the urine sample data was inconsistently collected by the subjects. Therefore, no adequate analysis of the urine samples could be performed and the bladder residence times were derived from the scintigraphic images only.

The organ-absorbed doses and the effective doses are summarized in Table 2, and the residence times in the Supplemental Table 1. The total-body absorbed dose in males was comparable to females (0.012 ± 0.004 vs. 0.011 ± 0.005 mSv/MBq; *p* = 0.97).

**Table 2:** Estimated organ radiation doses

Target organ	Organ dose (mSV/MBq)	
	Adult Male	Adult Female
Adrenals	7.03* ± 6.37*10^−4^	3.39*10^−4^ ± 3.47*10^−4^
Brain	1.55*10^−4^ ± 2.54*10^−4^	1.27*10^−5^ ± 3.95**10^−6^
Breasts		2.77*10^−4^ ± 9.37*10^−5^
Esophagus	1.38*10^−3^ ± 2.29*10^−3^	3.62*10^−4^ ± 1.06*10^−4^
Eyes	0.00*10^0^ ± 0.00*10^0^	0.00*10^0^ ± 0.00*10^0^
Gallbladder Wall	4.83*10^−4^ ± 6.04*10^−4^	2.14*10^−4^ ± 1.88*10^−4^
Left colon	2.28*10^−3^ ± 3.68*10^−3^	4.38*10^−4^ ± 2.52*10^−4^
Small intestine	5.16*10^−4^ ± 7.26*10^−4^	2.42*10^−4^ ± 4.42*10^−5^
Stomach Wall	5.02*10^−3^ ± 7.74*10^−3^	1.31*10 ^−^ ^3^ ± 6.57*10^−4^
Right colon	2.15*10^−3^ ± 3.69*10^−3^	4.31*10^−4^ ± 2.22*10^−4^
Rectum	9.23*10^−4^ ± 1.91*10^−3^	1.06*10^−4^ ± 5.08*10^−5^
Heart Wall	3.19*10^−4^ ± 5.21*10^−4^	7.06*10^−5^ ± 6.64*10^−6^
Kidneys	1.75*10^−3^ ± 2.92*10^−3^	1.48*10^−3^ ± 2.71*10^−3^
Liver	2.56*10^−3^ ± 1.14*10^−3^	2.01*10^−3^ ± 9.55*10^−4^
Lungs	3.43*10^−3^ ± 4.51*10^−3^	1.49*10^−3^ ± 6.94*10^−5^
Ovaries		1.91*10^−4^ ± 4.66*10^−5^
Pancreas	4.46*10^−4^ ± 6.81*10^−4^	1.70*10^−4^ ± 1.06*10^−4^
Prostate	1.81*10^−4^ ± 3.70*10^−4^	
Salivary Glands	5.02*10^−4^ ± 8.47*10^−4^	1.55*10^−5^ ± 6.43*10^−6^
Red Marrow	3.78*10^−3^ ± 7.34*10^−3^	5.61*10^−4^ ± 1.90*10^−4^
Osteogenic Cells	5.90*10^−4^ ± 1.17*10^−3^	6.92*10^−5^ ± 1.76*10^−5^
Spleen	1.50*10^−3^ ± 1.99*10^−3^	5.18*10^−4^ ± 2.91*10^−4^
Testes	5.56*10 ^−^ ^3^ ± 1.04*10^−2^	
Thymus	2.79*10^−4^ ± 5.54*10^−4^	4.15*10^−5^ ± 1.23*10^−5^
Thyroid	1.57*10^−3^ ± 2.98*10^−3^	1.12*10^−4^ ± 2.20*10^−5^
Urinary Bladder Wall	1.83*10^−3^ ± 3.14*10^−3^	6.46*10^−4^ ± 2.82*10^−4^
Uterus		2.88*10^−5^ ± 8.96*10^−6^
Total Body	1.12*10^−2^ ± 3.71*10^−3^	1.11*10 ^−^ ^2^ ± 5.36*10^−3^

Estimated radiation doses per organ as calculated using ICRP-89 models in OLINDA/EXM-based dosimetry

### Ex vivo binding of ^99m^Tc-fucoidan to human thrombus

**Fig. 3:**
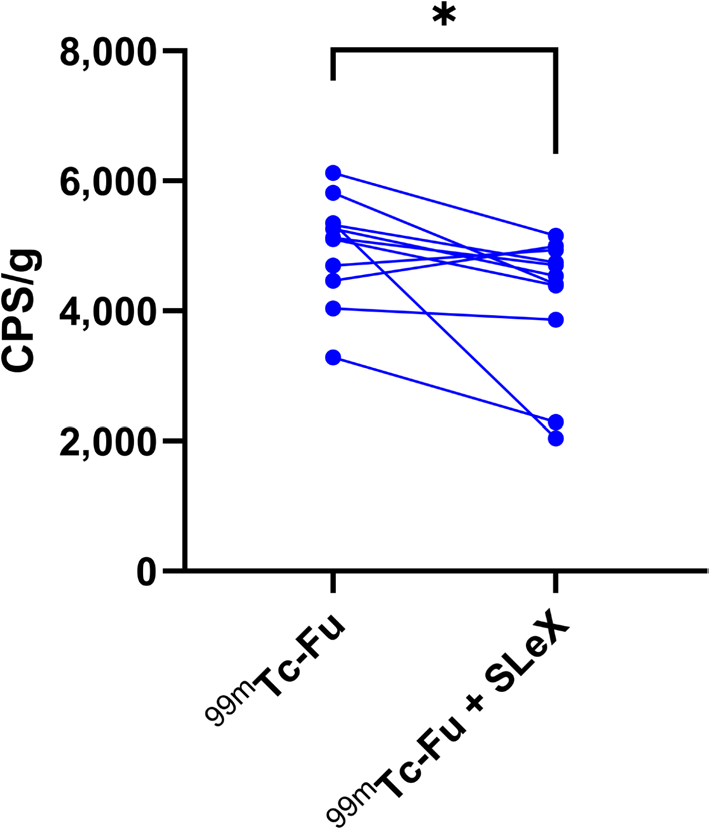
Ex vivo ^99m^Tc-fucoidan binding in human thrombi Binding of ^99m^Tc-fucoidan to human thrombi ex vivo, with and without blocking agent Sialyl Lewis X. ^99m^Tc-Fu indicates ^99^Tc-labeled fucoidan; and SLeX, Sialyl Lewis X

Ex vivo thrombi from all individuals remained stable throughout the tracer incubation and washing steps. The average ^99m^Tc-fucoidan uptake in ex vivo thrombus was 4961 ± 806 CPS/g, whereas the uptake was reduced by 16% after blocking with SLe^x^ (4188 ± 1061 CPS/g, *p* = 0.029, Fig. 3).

### ^99m^Tc-fucoidan uptake in deep vein thrombosis

**Fig. 4:**
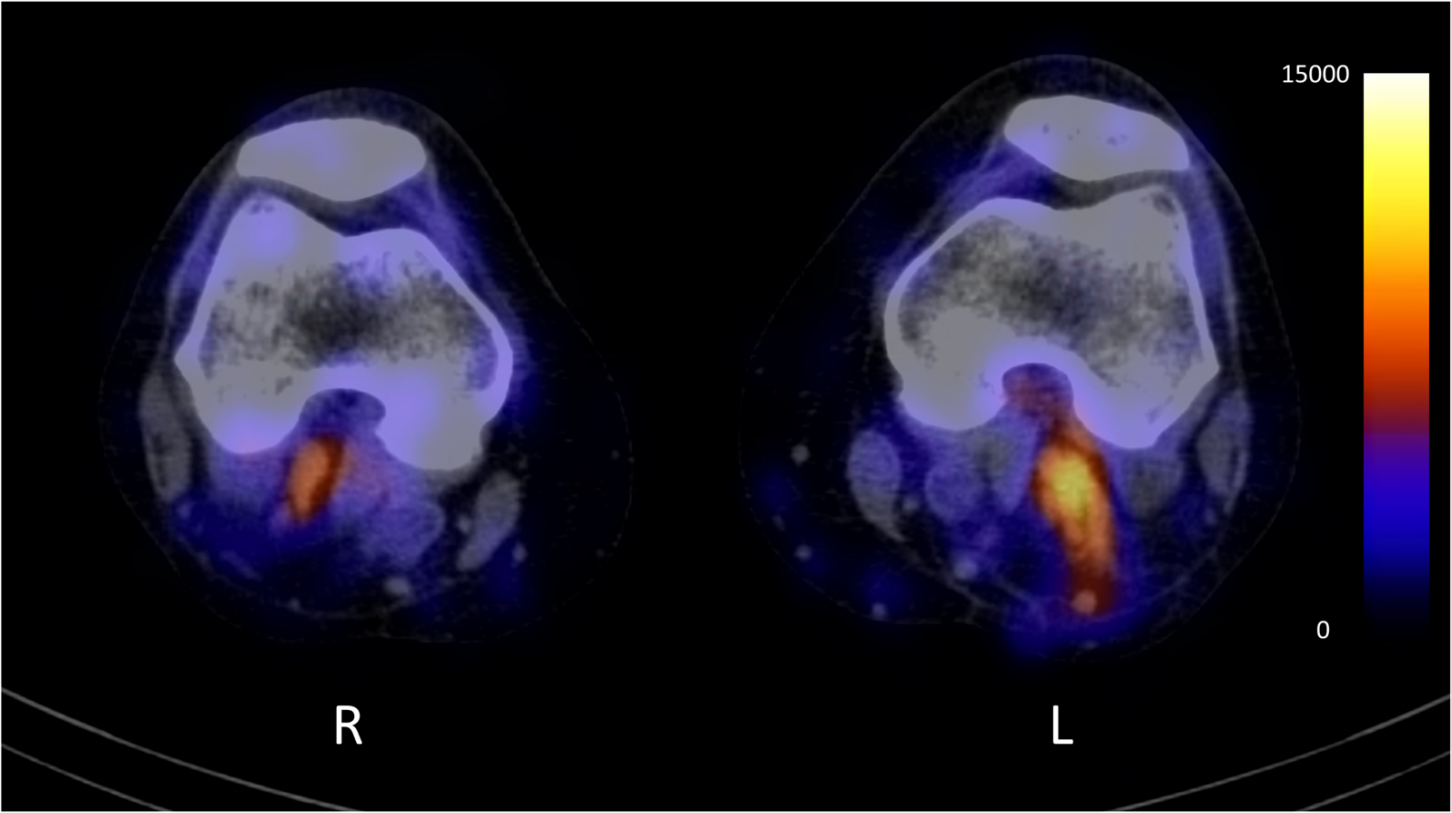
Focal uptake of ^99m^Tc-fucoidan in deep vein thrombosis of the left popliteal vein ^99m^Tc-fucoidan SPECT/CT image of a 50-year-old woman recently diagnosed with a deep vein thrombosis of the left popliteal vein. Focal uptake can be observed at the thrombus site. The scale bar is measured in absolute counts

High regional uptake of ^99m^Tc-fucoidan was observed in the left popliteal- and femoral vein in one patient (Fig. 4), corresponding to the site of the DVT on ultrasonography. Drawing a region of interest (ROI) around the thrombus and the unaffected contralateral vein, the maximum counts were 1.42 times as high in the thrombus. The scans of the remaining two patients showed no increased focal tracer uptake in the affected veins. In these patients the maximum counts in the thrombus were 0.84 and 0.93 times as high compared to the unaffected contralateral vein.

## Discussion

Here, we describe the first-in-human experience with a nuclear imaging agent for P-selectin, a transmembrane protein which plays an essential role in thrombus formation and also throughout all stages of the atherogenic process. Our study established clinical safety of ^99m^Tc-fucoidan and determined that tissue distribution and radiation exposure were appropriate for clinical use. Additionally, we demonstrated that ^99m^Tc-fucoidan binds to human thrombi ex vivo and in vivo.

Recently, we set up a GMP-grade radiosynthesis protocol for the production of ^99m^Tc-fucoidan [14]. There we showed that the GMP produced fucoidan had no side effects in rats after an intravenous administration of 1,000-fold excess of the planned clinical dose (40 µg/volunteer), and that fucoidan had no immune reactive properties in pigs [14]. In this study, we observed renal uptake of the radiotracer after intravenous injection of ^99m^Tc-fucoidan, followed by accumulation in the bladder and high activity in urine, indicating rapid renal excretion of ^99m^Tc-fucoidan. This is presumably due to the hydrophilic properties of low-molecular-weight fucoidan [14]. Moderate physiological uptake was observed in the liver which decreased rapidly over 24 h. Radioactivity measurements showed higher ^99m^Tc-fucoidan uptake in plasma as compared to whole blood, perhaps due to non-specific binding to proteins within plasma. Previous in vitro studies with whole human blood have demonstrated that the binding of ^99m^Tc-fucoidan to human platelets increases with the level of platelet activation and that ^99m^Tc-fucoidan further blocks binding sites for anti–P-selectin antibody in activated platelets, indicating a specific interaction between ^99m^Tc-fucoidan and P-selectin [10, 12].

Our findings are in agreement with prior biodistribution studies of radiolabeled fucoidan in animals, which also demonstrated rapid renal excretion and moderate liver uptake [4, 13]. Radioactive metabolites of ^99m^Tc-fucoidan may contribute to the high uptake observed in the liver, kidneys, and urinary bladder, but we did not assess the metabolism of ^99m^Tc-fucoidan in this study. Typically, the presence of free technetium indicating degradation of the tracer can be detected as radioactive accumulation in a thyroid gland and stomach. In the biodistribution study in rats [4] and in our study, the thyroid and stomach uptake of ^99m^Tc-fucoidan was comparable between every time point. Given the similarity in biodistribution patterns between rodents and humans observed in our study, the ^99m^Tc-fucoidan is likely metabolized in a similar manner in humans. However, the metabolism of ^99m^Tc-fucoidan in humans has to be confirmed in future studies.

Blood, brain and myocardial uptake of ^99m^Tc-fucoidan was low compared to other tracers used in cardiovascular imaging, such as ^18^F-FDG. This could offer significant advantages for the clinical integration of ^99m^Tc-fucoidan in cardiovascular research, as it eliminates the potential hindrance of myocardial spillover. Furthermore, the estimated total-body absorbed dose of ^99m^Tc-fucoidan (0.012 mSv/MBq) is within the range of ^18^F-FDG (0.015 mSv/MBq) [18] and other existing ^99m^Tc-labeled tracers (0.006–0.010 mSv/MBq) used for myocardial perfusion imaging [19] or detection of cardiac amyloidosis [20], bladder being the dose limiting organ. The bone marrow absorbed dose was calculated based on the source organ model, which may have underestimated the actual bone marrow dose. A single dose of ^99m^Tc-fucoidan did not exhibit any side effects in healthy volunteers.

Next, we examined the binding of ^99m^Tc-fucoidan to freshly prepared human thrombi from 11 volunteers. In vitro studies haves shown that ^99m^Tc-fucoidan binding to platelets is increased when the platelets are adhered to fibrinogen-coated plates resembling activation [13]. We extended this finding by demonstrating a reduction in ^99m^Tc-fucoidan uptake when the ex vivo thrombi were co-incubated with SLe^x^. Whilst a reduction of 16% does not indicate a complete blocking of P-selectin, it should be noted that significantly higher concentration of ^99m^Tc-fucoidan were utilized compared to those used in the clinical studies. This disparity in concentration likely contributed to a higher degree of non-specific uptake, thereby resulting in the less pronounced blocking effect observed. Moreover, the ex vivo incubation time of ^99m^Tc-fucoidan (1 h) was substantially long, since we desired to mimic the scanning time in clinical studies (2 h), although it may be that only a few minutes are needed to reach the maximal binding of ^99m^Tc-fucoidan/SLe^X^ to P-selectin. Previous in vitro studies [12] have used pharmacological agents to activate thrombus further that could have also increased the expression levels of P-selectin in our experiment and hence, facilitate the ligand binding and blocking effect.

Taking these limitations into account, the observed results are indicative of specific binding of ^99m^Tc-fucoidan to P-selectin expressed on human thrombi ex vivo. The inhibition of uptake in the presence of SLe^x^ underscores the importance of specific molecular interactions, likely involving selectin-based adhesion mechanisms, which are well-established in thrombus formation [21]. This finding not only highlights the unique binding properties of ^99m^Tc-fucoidan but also raises intriguing possibilities for its potential clinical applications in targeting thrombotic events with high precision. Almost a decade ago, focal vascular uptake of gallium-68-labeled fucoidan was detected in atherosclerotic apolipoprotein E-deficient mice by in vivo PET/MR. This uptake was also significantly reduced after blocking with SLe^x^ [4]. Furthermore, ^99m^Tc-fucoidan SPECT/CT was able to detect the presence of thrombi in abdominal aortic aneurysm and endocarditis rat models [13] paving the way for the evaluation of P-selecting imaging in clinical setting.

We envisioned that ^99m^Tc-fucoidan may bear clinical utility for the differentiation between recurrent and residual thrombosis, a common clinical problem [22]. Therefore, as proof of concept, we conducted ^99m^Tc-fucoidan SPECT imaging in three patients with a recently diagnosed DVT. Focal tracer uptake was clearly visible in one DVT case. However, the remaining 2 scans showed no focal uptake of ^99m^Tc-fucoidan in the affected veins. This apparent discrepancy may have several explanations. First, it could be that detection by ^99m^Tc-fucoidan is predominantly present on fresh thrombi strongly expressing P-selectin and without having a tight fibrin cap. Indeed, the time varied between the onset of deep vein thrombosis and admission to the hospital among the patients. Second, thrombi are heterogeneous in nature and include varying degrees of fibrin, activated platelets, leukocytes, erythrocytes, and neutrophil extracellular traps. Thrombi biological components also vary among the arterial, venous, and microcirculatory compartments. To our knowledge, there has been one other ^99m^Tc-labeled tracer investigated for the detection of DVT [23]. ^99m^Tc-apcitide is a synthetic polypeptide, which binds to glycoprotein IIb/IIIa receptors, expressed also on activated platelets. In 2008, Dunzinger et al. demonstrated that with ^99m^Tc-apcitide they were able to identify acute clot formation in 14 out of 16 patients with DVT, up to 17 days after onset of clinical symptoms. However, this tracer failed to detect pulmonary embolism.

However, with the given discrepancy that ^99m^Tc-fucoidan was able to detect only 1 out of 3 DVTs in this proof of concept study, further clinical development of ^99m^Tc-fucoidan as an imaging approach for patients with DVT is not warranted. Other potential clinical applications for this technetium-99m-labeled P-selectin tracer where endothelial activation plays a critical role may be considered, such as imaging of arterial thrombosis, large vessel vasculitis, myocarditis, or vascular inflammation due to rheumatoid arthritis or novel anti-cancer therapies.

Limitations of this study include the small sample size. While a statistically significant outcome was achieved in the in vitro experiment, caution should be exercised when interpreting these data. Additionally, despite the recent advances in SPECT technology, SPECT has a lower spatial resolution compared to other imaging modalities such as PET and MRI, that may be a challenge in pinpointing the exact location of tracer binding within the small structures like vasculature.

## Conclusion

^99m^Tc-fucoidan, the P-selectin targeting SPECT tracer has a favorable safety profile combined with an acceptable estimated total-body absorbed dose. Nonetheless, the in vivo results do not support further clinical investigation of ^99m^Tc-fucoidan as an imaging modality for DVT. Other clinical implementations of a technetium− 99m-labeled P-selectin tracer should be considered.

## Supplementary Information

Additional file 1.

## Data Availability

The datasets used and/or analysed during the current study are available from the corresponding author on reasonable request.
