# Supplementary material for: First-in-human study of 99mTc-labeled fucoidan, a SPECT tracer targeting P-selectin
Source: EJNMMI Res. 2024 Nov 19;14:112. doi: 10.1186/s13550-024-01173-8 (PMC11576749; doi:10.1186/s13550-024-01173-8)
Supplement: Supplementary file 1 — Additional file 1. [file 13550_2024_1173_MOESM1_ESM.docx]

**Supplementary Information**

**Supplementary Methods**

To limit overlapping organs from distorting tracer uptake measurements, following strategies were applied (1): For paired organs, such as the kidneys and lungs, the activity was quantified in one of the organs for which there was no overlap with other organs and the number of counts was doubled to obtain the total radioactivity in both organs. For non-paired organs, we chose to draw a ROI over the region of the organ that has no overlap in scans where there is overlap, counted the number of pixels, noted the average count per pixel, used a ROI from another image in which there was no apparent overlap and the whole organ was clearly visible, counted the number of pixels in a larger ROI drawn on this image, and then multiplied the count per pixel from the first image by the number of pixels in the second image in order to estimate the total radioactivity from the organ in the first image.

**Supplementary Table 1.** The number of disintegrations (residence times) in source organs.

| Target Organ | Mean | SE |
| --- | --- | --- |
| Brain | 0.034 ± 0.008 | 0.002 |
| Small intestine | 0.622 ± 0.098 | 0.031 |
| Heart contents | 0.077 ± 0.020 | 0.006 |
| Left kidney | 0.151 ± 0.042 | 0.013 |
| Right kidney | 0.256 ± 0.122 | 0.038 |
| Liver | 3.158 ± 0.979 | 0.310 |
| Left lung | 0.245 ± 0.062 | 0.020 |
| Right lung | 0.178 ± 0.034 | 0.011 |
| Muscle | 0.022 ± 0.007 | 0.002 |
| Spleen | 0.311 ± 0.141 | 0.045 |
| Testes | 0.335 ± 0.557 | 0.250 |
| Urinary bladder contents | 0.302 ± 0.130 | 0.041 |
| Total body | 7.440 ± 0.275 | 0.092 |

1. Stabin MG, Wendt RE, Flux GD. RADAR Guide: Standard Methods for Calculating Radiation Doses for Radiopharmaceuticals, Part 1—Collection of Data for Radiopharmaceutical Dosimetry. Journal of Nuclear Medicine 2022;63:316.
